# Changes in Monoaminergic Neurotransmission in an Animal Model of Osteoarthritis: The Role of Endocannabinoid Signaling

**DOI:** 10.3389/fnmol.2018.00466

**Published:** 2018-12-20

**Authors:** Jakub Mlost, Agnieszka Wąsik, Jerzy Tadeusz Michaluk, Lucyna Antkiewicz-Michaluk, Katarzyna Starowicz

**Affiliations:** Department of Neurochemistry, Institute of Pharmacology, Polish Academy of Sciences, Kraków, Poland

**Keywords:** osteoarthritis, chronic pain, catecholamines metabolism, brain structures, reward system, hypodopaminergia, endocannabinoid system

## Abstract

Chronic pain is a main symptom of osteoarthritis (OA). Moreover, a high percentage of OA patients suffer from mental health problems. The endocannabinoid (EC) system has attracted attention as an emerging drug target for pain treatment together with its activity on the mesolimbic reward system. Understanding the circuits that govern the *reward* of *pain relief* is crucial for the search for effective analgesics. Therefore, we investigated the role of the EC system on dopamine (DA) and noradrenaline (NA) in an animal model of OA-related chronic pain. OA rats exhibited significant decreases in DA metabolism in the nucleus accumbens (NAc), striatum (STR) and hippocampus (HC). NA metabolism was also significantly decreased by chronic pain in OA rats; however, this disruption was limited to the frontal cortex (FCx) and HC. URB597 (an inhibitor of EC metabolism) treatment completely reversed the decreased DA metabolism, especially in the brain reward system and the HC. Furthermore, administration of URB597 normalized the impairment of NA activity in the HC but potentiated the decreased NA levels in the FCx. Our results demonstrated that chronic pain in OA rats was reflected by the inhibition of mesolimbic and mesocortical dopaminergic transmission, and may indicate the pro-pain role of NA in the FCx. The data provide understanding about changes in neurotransmission in chronic pain states and may explain the clinical improvement in perceived life quality following cannabinoid treatment. Additional mechanistic studies in preclinical models examining the intersection between chronic pain and reward circuits may offer new approaches for improving pain therapy.

## Introduction

According to the International Association for the Study of Pain (IASP), pain is defined as a complicated phenomenon compromising an unpleasant sensory and emotional experience associated with actual or potential tissue damage. Pain is a crucial evolutionary mechanism designed for the survival of organisms. However, long-lasting noxious stimulation is no longer serving its evolutionary role and turning into an unbearable experience.

Pain chronification involves structural and functional changes in the central nervous system (Apkarian et al., 2013; Mansour et al., 2013). Accumulating evidence has demonstrated that structures engaged in reward circuits are also involved in the transition from acute to chronic pain (Baliki et al., 2012; Vachon-Presseau et al., 2016). Recent work from Martikainen et al. (2015) showed a significant decrease in dopamine (DA) signaling in the ventral tegmental area, in chronic pain patients, and this decrease was negatively correlated with pain sensitivity.

Moreover, there is a high comorbidity between chronic pain and mental health problems related to dopaminergic signaling dysfunction. For instance, Nazarinasab reported in 2017, that more than half of osteoarthritic patients suffered from mental health problems, such as obsessive-compulsive disorder, depression or anxiety (Leite et al., 2011; Nazarinasab et al., 2017). Additionally, the risk of suicide is considerably high among chronic pain patients (Hassett et al., 2014). Strikingly, even a higher number (up to 80%) of major depression or Parkinson’s disease patients report comorbid pain conditions (Leuchter et al., 2010; Skogar and Lökk, 2016; Young Blood et al., 2016). Further research showed that Parkinson’s disease patients report higher pain ratings while off their treatment (levodopa, orally bioavailable precursor of DA; Nebe and Ebersbach, 2009).

Current treatment of chronic pain is based on opioid drugs that exertpronounced antinociceptive effects and have a strong rewarding effect that often leads to addiction and a phenomenon called opioid-induced hyperalgesia. Alternatively, targeting the endocannabinoid (EC) system could become a novel treatment strategy for chronic pain patients. EC consists of ECs, enzymes responsible for their metabolism and receptors, CB1 and CB2, both of which are highly implicated in pain and reward processing. Targeting enzymes involved in the degradation of ECs seems to be a promising treatment strategy as ECs are produced “on demand” and therefore, inhibition of degradative enzymes (such as fatty acid amide hydrolase, FAAH) should be devoid of unwanted side effects as the increase of EC levels should be limited specifically to disease-affected tissues.

The aim of our research was to evaluate the DA and noradrenaline (NA) concentrations and the rate of their metabolism in mesolimbic and mesocortical brain structures in response to osteoarthritis (OA)-related pain, and to monitor the effect of EC system activation.

## Materials and Methods

### Animals

Male Wistar rats (Charles River, Hamburg, Germany), initially weighing 225–250 g, were used for all experiments. Animals were housed five per cage under a standard 12 h/12 h light/dark cycle (lights on at 06:00 h) with food and water available *ad libitum*. This study was carried out in accordance with the principles of the Basel Declaration and the recommendations of the IASP and 3R policy [IASP Guidelines for the Use of Animals in Research; International Association for the Study of Pain (IASP), 2018]. The protocol was approved by the Local Ethics Committee of the Institute of Pharmacology (Cracow, Poland, approval number 125/2018). Care was taken to implement the “3 Rs” rule (replacement, reduction and refinement) to reduce the number of animals used and their suffering during the experiments.

### OA Induction

The rats were deeply anesthetized with 5% isoflurane (Forane^®^, Baxter Healthcare Corporation, Deerfield, IL, USA) in 100% O_2_ (3 L/min). The skin covering the right knee joint was shaved and swabbed with 100% ethanol. A 27-gauge needle was introduced into the joint cavity through the patellar ligament, and 50 μl containing 1 mg of sodium monoiodoacetate (MIA) in 0.9% saline was injected intra-articular to induce OA-like lesions.

### Drugs

MIA, dimethyl sulfoxide (DMSO), and Kolliphor EL were obtained from Sigma-Aldrich (Poznan, Poland). URB-597 was obtained from Tocris Bioscience (Bristol, UK); URB597 was dissolved in 5% DMSO, 5% ethanol, 5% Kolliphor EL and 85% saline at a 1 mg/ml concentration. MIA was dissolved in 50 μl of 0.9% saline.

### *Ex vivo* Biochemical Studies

At day 28 post MIA injection, rats were treated with 1 mg/kg of URB597 i.p. and 1 h later sacrificed by decapitation. Brains were rapidly removed and dissected on an ice-cold glass plate, and the following structures were isolated: striatum (STR), nucleus accumbens (NAc), hippocampus (HC) and frontal cortex (FCx). The obtained tissue was frozen on solid CO_2_ (−70°C) and stored until used for biochemical assays. DA and its final metabolite homovanillic acid (HVA), NA and its extracellular metabolite normetanephrine (NM), were assayed by high-performance liquid chromatography (HPLC) with electrochemical detection. The tissue samples were weighed and homogenized in ice-cold 0.1 M perchloroacetic acid containing 0.05 mM ascorbic acid. After centrifugation (10,000× *g*, 5 min), the supernatants were filtered through RC 58 0.2 μm cellulose membranes (Bioanalytical Systems, West Lafayette, IN, USA). The mobile phase consisted of 0.05 M citrate-phosphate buffer (pH 3.5), 0.1 mM EDTA, 1 mM sodium octyl sulfonate and 3.5% methanol. The HP 1050 chromatograph (Hewlett-Packard, Golden, CO, USA) was equipped with a C18 column. The flow rate was maintained at 1 ml/min. DA, NA and their metabolites were quantified by peak height compared with standards run on the day of the analysis. Each group comprised 5–8 animals.

### Statistical Analysis

One-way analysis of variance (ANOVA) was used to analyze the results from the neurochemical studies. Differences between the control and experimental groups were assessed using Duncan’s *post hoc* tests. The data were considered statistically significant when *P* < 0.05.

The total metabolism rate for DA was assessed from the ratio of the final DA metabolite HVA concentration to DA concentration and expressed as the catabolic rate index [HVA]/[DA] × 100; analogously, the rate of NA metabolism was expressed as the ratio of the extraneuronal NA metabolite concentration [NM] to [NA] × 100. The indices were calculated using concentrations from individual tissue samples (Antkiewicz-Michaluk et al., 2001).

## Results

### The Effect of URB597 on OA-Produced Decreases in Dopaminergic Neurotransmission in the Brain

#### DA Concentrations

**Figure 1:**
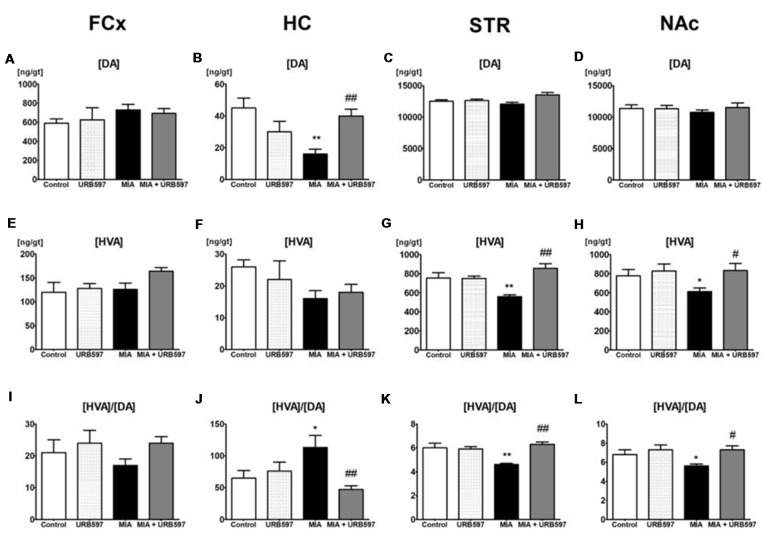
The effect of URB597 on osteoarthritis (OA)-produced decreases in dopaminergic neurotransmission in the investigated brain structures; **(A,E,I)** frontal cortex (FCx), **(B,F,J)** hippocampus (HC), **(C,G,K)** striatum (STR) and **(D,H,L)** nucleus accumbens (NAc). Samples were collected 28 days after OA induction, 1 h after i.p. administration of URB597 or vehicle. Data are presented as the mean ± SEM and represent normalized averages derived from 6 to 10 samples for each group. The results were analyzed by means of one-way analysis of variance (ANOVA), followed by Duncan’s *post hoc* tests. Statistical significance: *P < 0.05, **P < 0.01 vs. Control group; ^#^*P* < 0.05, ^##^*P* < 0.01 vs. OA group. One-way ANOVA indicated a significant effect of OA on the rate of dopamine (DA) metabolism in STR (*F*_(3,22)_ = 13.89; *P* < 0.00002) and NAc (*F*_(3,21)_ = 4.18; *P* < 0.01); DA concentration in STR (*F*_(3,22)_ = 4.32; *P* < 0.01) and HC (*F*_(3,22)_ = 6.96; *P* < 0.001) and homovanillic acid (HVA) concentration in STR (*F*_(3,22)_ = 13.10; *P* < 0.00004) and NAc (*F*_(3,21)_ = 3.48; *P* < 0.03).

One-way ANOVA showed significant effects of OA on DA concentrations in the STR and HC (Figures 1B,C and Supplementary Table S1) but no effects were observed in FCx and NAc (Figures 1A,D). Duncan’s *post hoc* tests indicated that MIA significantly decreased the level of DA in the HC (approximately 60%), and URB597 administration (1 mg/kg, i.p.) completely antagonized this effect and restored DA concentration to the control value (Figure 1B).

#### HVA Concentrations (The Final Metabolite of DA)

One–way ANOVA demonstrated a significant effect of OA on HVA concentrations in the STR and NAc (Supplementary Table S1) but no effects were observed in FCx and HC (Figures 1E,F). Duncan’s *post hoc* tests indicated that MIA significantly decreased HVA concentrations in the STR (approximately 30%) and NAc (approximately 20%). The same analysis showed that administration of URB597 completely antagonized the decreases in HVA concentration in the OA group (Figures 1G,H).

#### Rates of DA Metabolism

One-way ANOVA indicated a significant effect of OA on the rate of DA metabolism, presented as the ratio [HVA]/[DA] in the STR and NAc (Figures 1K,L and Supplementary Table S1) but no effects were observed in FCx and HC (Figures 1I,J). Duncan’s *post hoc* tests revealed that OA significantly decreased the rate of DA metabolism in both investigated structures). URB597 significantly restored the rate of DA metabolism in both structures to the control values (Figures 1K,L).

### The Effect of URB597 on OA-Produced Changes in NA Transmission in the Brain

#### NA Concentrations

**Figure 2:**
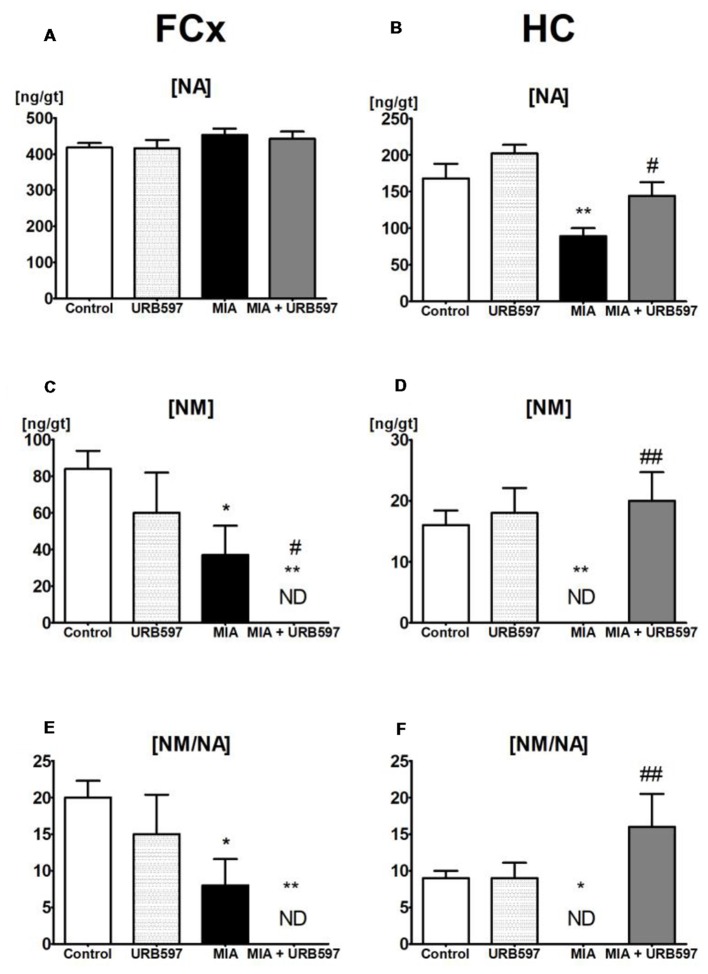
The effect of URB597 on OA-produced decreases in noradrenergic neurotransmission in the investigated brain structures; **(A,C,E)** FCx and **(B,D,F)** HC. Samples were collected 28 days after OA induction, 1 h after i.p. administration of URB597 or vehicle. Data are presented as the mean ± SEM and represent normalized averages derived from 6 to 10 samples for each group. The results were analyzed by means of one-way ANOVA, followed by Duncan’s *post hoc* tests. Statistical significance: *P < 0.05, **P < 0.01 vs. Control group; ^#^*P* < 0.05, ^#^^#^*P* < 0.01 vs. OA group. One-way ANOVA indicated a significant effect of OA on the rate of noradrenaline (NA) metabolism in FCx (*F*_(3,22)_ = 5.69; *P* < 0.004) and HC (*F*_(3,22)_ = 6.88; *P* < 0.001); NM concentration in FCx (*F*_(3,22)_ = 5.66, *P* < 0.004) and HC (*F*_(3,22)_ = 8.92, *P* < 0.0004) and NA concentration in the HC (*F*_(3,22)_ = 9.99, *P* < 0.0002).

One-way ANOVA showed a significant effect of OA on the NA concentration in the HC (Supplementary Table S2) and Duncan’s *post hoc* test revealed a decrease in NA concentration (approximately 45%) in the OA group (Figure 2B), however it was not affected in FCx (Figure 2A). URB597 administration to OA rats significantly antagonized the decrease in NA in the HC (Figure 2B).

#### NM Concentrations (The Extraneuronal Metabolite of NA)

ANOVA demonstrated a significant effect of OA on the concentrations of NM (Supplementary Table S2), which may be a good intermediate indicator of NA release in the FCx and HC. Duncan’s *post hoc* tests showed that OA induced a strong decrease in NM concentrations in the HC (approximately 100%) as well as in the FCx (approximately 60%), and URB597 administration restored it to the control level in the HC but intensified the decrease in NM in the FCx (Figures 2C,D).

#### Rates of NA Metabolism

One-way ANOVA indicated a significant effect of OA on rates of NA metabolism, as shown by the ratio [NM]/[NA] in the FCx and HC (Supplementary Table S2). Duncan’s *post hoc* tests revealed that OA significantly decreased rates of NA metabolism in the FCx, and completely inhibited NA release in the HC. URB597 administration to OA-rats completely antagonized the decreased NA metabolism in the HC and significantly intensified the rate of NA metabolism in the FCx (Figures 2E,F).

## Discussion

Our results showed that the chronic pain state due to OA was reflected by significant inhibition of dopaminergic transmission. This result may reflect the high depression comorbidity observed in OA patients (Salamone et al., 2006, 2016). Furthermore, it is well known that dopaminergic transmission within NAc is an important factor in mediating the suppression of tonic pain (Altier and Stewart, 1999; Wood, 2008) and lesions within the mesostriatal pathway lead to the development of mechanical hypersensitivity (Takeda et al., 2005). URB597 treatment was able to reverse disruptions in dopaminergic signaling within the NAc in OA-rats; importantly, it had no effect on DA signaling in healthy rats. This not only reflects the antinociceptive potential of FAAH inhibitors (Jayamanne et al., 2006; Russo et al., 2007) but also suggests that an increase in EC tone may elicit rewarding and antidepressive effects, but only in impaired animals. Indeed, most recent findings have confirmed that inhibition of FAAH improved depressive-like behaviors induced by neuropathic pain. Interestingly, the peripherally restricted FAAH inhibitor failed to induce antidepressive effects despite retaining antinociceptive potential (Jiang et al., 2018). In addition, DA also exerts antinociceptive effects within the HC (Shamsizadeh et al., 2013; Reisi et al., 2014). Indeed, we also observed a decrease in DA levels in the HC following OA induction, which was reversed by URB597 treatment. In conclusion, URB597 is able to restore DA depletion due to OA-related chronic pain without affecting DA transmission in healthy animals.

Another significant factor in the top-down control of pain sensation is NA, which can exert opposite effects on pain transmission. For example, NA release mediates the inhibition of spinal nociceptive transmission by the descending pathways originating in the brainstem (Millan, 2002). Similar to DA, NA has been implicated as an antinociceptive neurochemical within the HC (Jin et al., 2014). In contrast, in chronic pain conditions, NA release in the FCx is actually responsible for pain generation (Taylor and Westlund, 2017). In our research, we observed a decline in NA release in both of these structures. URB597 was able to reverse NA levels in the HC, however it also caused a complete depletion of NA release in the FCx, without affecting NA concentration. These results are probably related to the analgesic potential of URB597.

In summary, our results demonstrated that increasing EC tone, counteracts the hypodopaminergic state in OA animals without affecting healthy animals. However, these results are not in line with the clinical research that has shown a lack of efficacy of FAAH inhibitors in reducing pain in OA (Huggins et al., 2012) and symptoms of depression (Sanofi, 2013). It is not known what causes this discrepancy between the very promising preclinical data and the lack of FAAH inhibitor efficacy in the clinic. Off-target activity of AEA or URB597 itself could play an important role. For example, AEA is also a TRPV1 agonist (Starowicz et al., 2013) and thus it may mediate pro-nociceptive effects as well. Additionally, the biological actions of FAAH extend far beyond the termination of anandamide signaling. Broad changes in lipid levels may arise as a result of the redundancy of EC inactivation (Piscitelli and Di Marzo, 2012). Moreover, in light of the present study it is important to note poor specificity of URB597 (Zhang et al., 2007), which has been shown to upregulate tyrosine hydrolase independently FAAH inhibition (Bosier et al., 2013). This study was preliminary and therefore, the exact mechanism of action was not established and off-target activity of URB597 may not be excluded. Further experiments with use of more specific FAAH inhibitors and cannabinoid receptor antagonists are required.

## Data Availability

The raw data supporting the conclusions of this manuscript will be made available by the authors, without undue reservation, to any qualified researcher.

## Author Contributions

JM was responsible for drug treatment, participated in the study design, data interpretation and preparation of manuscript. AW performed the experiment and collected the data. JTM conducted the statistical analyses. LA-M participated in study design, data interpretation and preparation of manuscript. KS conceived and designed the study and was involved in interpretation of the data, preparation of the manuscript and revisions for intellectual content.

## Conflict of Interest Statement

The authors declare that the research was conducted in the absence of any commercial or financial relationships that could be construed as a potential conflict of interest.
