# Supplementary material for: Changes in Monoaminergic Neurotransmission in an Animal Model of Osteoarthritis: The Role of Endocannabinoid Signaling
Source: Front Mol Neurosci. 2018 Dec 20;11:466. doi: 10.3389/fnmol.2018.00466 (PMC6306412; doi:10.3389/fnmol.2018.00466)
Supplement: Supplementary file 1 [file Table_1.docx]

Supplementary Material

*Changes in monoaminergic neurotransmission in an animal model of osteoarthritis: the role of endocannabinoid signaling*

**Jakub Mlost**^1^**, Agnieszka Wąsik**^1^**, Jerzy Michaluk**^1^**, Lucyna Antkiewicz-Michaluk**^1^***, Katarzyna Starowicz**^1^*****

*** Correspondence: Professor Lucyna Antkiewicz-Michaluk**

**Tel +48 12 6623203; email:** [**antkiew@if-pan.krakow.pl**](mailto:antkiew@if-pan.krakow.pl)

**Professor Katarzyna Starowicz**

**Tel +48 12 6623206, email:** [**starow@if-pan.krakow.pl**](mailto:starow@if-pan.krakow.pl)

# Supplementary Data

**Table 1.** The effect of URB597 on OA-produced decreases in dopaminergic neurotransmission in the investigated brain structures.

| **FCx** | | | | |
| --- | --- | --- | --- | --- |
| **Treatment** | **N** | **DA**  (ng/g t) | **HVA**  (ng/g t) | **[HVA]/[DA]** |
| Control | 5 | 591±43 | 120±21 | 21±4 |
| URB597 | 6 | 626±126 | 128±10 | 24±4 |
| OA | 8 | 730±57 | 126±13 | 17±2 |
| OA+URB597 | 7 | 694±50 | 164±8**^*^** | 24±2 |
| *F* |  | *F_(3/22)_=0,69*  *NS* | *F_(3/22)_=2,37*  *NS* | *F_(3/22)_=1,31*  *NS* |
| **HC** | | | | |
| Control | 5 | 45±6,2 | 26±2,2 | 65±12 |
| URB597 | 6 | 30±6,6 | 22±5,9 | 76±14 |
| OA | 8 | 16±3,1**^**^** | 16±2,5 | 113±19**^*^** |
| OA+URB597 | 7 | 40±4,2**^##^** | 18±2,5 | 47±6**^##^** |
| *F* |  | *F_(3/22)_=6,96*  *P<0,001* | *F_(3/22)_=1,64*  *NS* | *F_(3/22)_=4,18*  *P<0,01* |
| **STR** | | | | |
| Control | 5 | 12527±261 | 754±55 | 6,0±0,4 |
| URB597 | 6 | 12673±228 | 750±25 | 5,9±0,2 |
| OA | 8 | 12073±329 | 558±22**^**^** | 4,6±0,1**^**^** |
| OA+URB597 | 7 | 13559±358 | 857±47^##^ | 6,3±0,2^##^ |
| *F* |  | *F_(3/22)_=4,32*  *P<0,01* | *F_(3/22)_=13,10*  *P<0,00004* | *F_(3/22)_=13,89*  *P<0,00002* |
| **NAc** | | | | |
| Control | 5 | 11376±578 | 778±66 | 6,8±0,5 |
| URB597 | 5 | 11339±555 | 828±73 | 7,3±0,5 |
| OA | 8 | 10766±393 | 613±36* | 5,6±0,2* |
| OA+URB597 | 7 | 11560±722 | 843±73**^#^** | 7,3±0,4**^#^** |
| *F* |  | *F_(3/21)_=0,43*  *NS* | *F_(3/21)_=3,48*  *P<0,03* | *F_(3/21)_=4,18*  *P<0,01* |

Legends: Brain structures: FCx – frontal cortex; HC – hippocampus; STR – striatum; NAc – nucleus accumbens. The Control group received intra-articular injections of 0.9% saline to the right knee, and after 28 days, the animals were decapitated. The URB597 group was intraperitoneally (i.p.) administered a dose of 1 mg/kg to the control rats, and 1 h later the rats were decapitated. The OA group (symptom of osteoarthritis) developed chronic pain following intra-articular injection of 1 mg sodium monoiodoacetate (MIA) to the right knee, and 28 days later, the rats were decapitated. The OA+URB597 group, at day 28 post MIA injections, was treated with URB597 at a dose of 1 mg/kg i.p. and 1 h later decapitated. The data are the means ± S.E.M. The results were analyzed by means of one-way ANOVA, followed by Duncan’s post hoc tests. Statistical significance: *P < 0.05, **P < 0.01 vs. Control group; ^#^P < 0.05, ^##^P < 0.01 vs. OA group.

**Table 2.** The effect of URB597 on OA-induced changes in noradrenergic transmission in the frontal cortex and hippocampus of the rat brain.

| **FCx** | | | | | | | | |
| --- | --- | --- | --- | --- | --- | --- | --- | --- |
| **Treatment** | | N | | **NA (ng/g t)** | | **NM (ng/g t)** | | **[NM]/[NA]** |
| Control | | 5 | | 418±13 | | 84±10 | | 20±2,3 |
| URB597 | | 6 | | 416±23 | | 60±22 | | 15±5,4 |
| OA | | 8 | | 453±18 | | 37±16^*^ | | 8±3,6^*^ |
| OA+URB597 | | 7 | | 442±21 | | 0,0**^**#^** | | 0,0**^**^** |
| *F* | |  | | *F_(3/22)_=0,87*  *NS* | | *F_(3/22)_=5,66*  *P<0,004* | | *F_(3/22)_=5,69*  *P<0,004* |
| **HC** | | | | | | | | |
| Control | 5 | | 168±20 | | 16±2,4 | | 9±1,0 | |
| URB597 | 6 | | 202±12 | | 18±4,1 | | 9±2,1 | |
| OA | 8 | | 89±11**^**^** | | 0,0**^**^** | | 0,0**^*^** | |
| OA+URB597 | 7 | | 144±19**^#^** | | 20±4,7**^##^** | | 16±4,5**^##^** | |
| *F* |  | | *F_(3/22)_=9,99*  *P<0,0002* | | *F_(3/22)_=8,92*  *P<0,0004* | | *F_(3/22)_=6,88*  *P<0,001* | |

Legends: Brain structures: FCx – frontal cortex; HC – hippocampus. The Control group received intra-articular injections of 0.9% saline to the right knee, and after 28 days, the animals were decapitated. The URB597 group was intraperitoneally (i.p.) administered a dose of 1 mg/kg to the control rats, and 1 h later the rats were decapitated. The OA group (symptom of osteoarthritis) developed chronic pain following intra-articular injection of 1 mg sodium monoiodoacetate (MIA) to the right knee, and 28 days later, the rats were decapitated. The OA+URB597 group, at day 28 post MIA injections, was treated with URB597 at a dose of 1 mg/kg i.p. and 1 h later decapitated. The data are the means ± S.E.M. The results were analyzed by means of one-way ANOVA, followed by Duncan’s post hoc tests. Statistical significance: *P < 0.05, **P < 0.01 vs. Control group; ^#^P < 0.05, ^##^P < 0.01 vs. OA group.
